# Simultaneous integrated boost in locally advanced cervical cancer patients ineligible for brachytherapy: Dosimetric comparison of VMAT versus helical tomotherapy

**DOI:** 10.1002/acm2.70418

**Published:** 2025-12-18

**Authors:** Muhammed İsmet Sekmen, Timur Koca, Durmuş Ali Çetmi, Yılmaz Bilek, Rahmi Atıl Aksoy, İsmail Hakkı Sarpün, Aylin Fidan Korcum, Nina Tunçel

**Affiliations:** ^1^ Department of Radiation Oncology Akdeniz University School of Medicine Antalya Turkey; ^2^ Department of Radiation Oncology Izmir City Hospital Izmir Turkey

**Keywords:** cervical cancer, radiotherapy, simultaneous integrated boost, tomotherapy, VMAT

## Abstract

**Objective:**

This retrospective, exploratory study evaluated the dosimetric comparison and feasibility of simultaneous integrated boost (SIB) delivered with volumetric modulated arc therapy (VMAT) and helical tomotherapy (HT) in patients with locally advanced cervical cancer ineligible for brachytherapy (BRT).

**Methods:**

This exploratory study involved dosimetric treatment planning based on data from 10 patients diagnosed with locally advanced cervical cancer. SIB plans delivering 77.5 Gy to the primary tumor and 45 Gy to elective regions in 25 fractions were generated using VMAT and HT techniques. SIB plans were created using the VMAT technique in the Monaco 5.51 treatment planning system (TPS) (Elekta, Stockholm, Sweden) and the HT technique in the Precision 3.3.1 TPS (Accuray, Madison, Wisconsin, USA). Planning target volume (PTV) coverage, organ‐at‐risk (OAR) doses, and dose–volume metrics of SIB plans were compared between the VMAT and HT techniques using appropriate statistical tests.

**Results:**

The median PTV77.5 volume was 83.4 cc (range: 14.9–218.2 cc). For PTV77.5, HT yielded a higher mean dose (79.52 Gy vs. 77.54 Gy, *p* = 0.001) and a superior conformity index (0.81 vs. 0.71, *p *< 0.001). Bladder dose metrics were significantly lower with HT, including V53.28 Gy (5.66% vs. 10.31%; *p *= 0.006) and D_2cc_ (66.47 Gy vs. 70.60 Gy;* p *< 0.05), while bowel V36.89 Gy volume was also reduced (94.03 cc vs. 115.13 cc; *p *= 0.038). No statistically significant differences were observed in rectal, sigmoid, or femoral head dose parameters. Beam‐on time was significantly longer for HT than for VMAT (11.0 min vs. 3.4 min, *p *= 0.005).

**Conclusions:**

BRT remains the standard of care for cervical cancer. For patients ineligible for BRT, SIB is dosimetrically feasible; in this exploratory planning study, HT‐SIB provided better target coverage and OAR sparing than VMAT‐SIB. Prospective multicenter validation is necessary before wider clinical adoption.

## 1 INTRODUCTION

Cervical cancer is the fourth most diagnosed cancer among women under 45 years of age and the third leading cause of cancer‐related mortality in women globally.^1^ Although the incidence of cervical cancer has been steadily declining in developed countries due to the implementation of Pap smear screening programs and the widespread use of prophylactic vaccines against human papillomavirus (HPV), it continues to be a major cause of morbidity and mortality in resource‐limited and underdeveloped regions.^2^, ^3^ The disease stage guides the treatment approach for cervical cancer. In cases of locally advanced cervical cancer, classified as stages IB3 to IVA according to the International Federation of Gynecology and Obstetrics (FIGO) staging system, the standard treatment involves concurrent chemoradiotherapy, followed by brachytherapy (BRT).^4^

In cervical cancer, increasing the tumor dose in radiotherapy enhances treatment effectiveness, but it also raises the risk of late adverse effects on surrounding organs at risk (OARs), such as the bladder, rectum, sigmoid colon, and intestines. Adding BRT to external beam radiotherapy (EBRT) reduces this risk by offering better protection to these organs while improving treatment outcomes. Compared to EBRT, BRT provides a distinct advantage due to its dose distribution, characterized by a low integral dose and a steep dose gradient.^5^ However, the BRT technique also has certain limitations.^6^ First, despite its association with optimal treatment outcomes, high‐quality, volumetric image‐guided BRT is available in only a small number of clinics worldwide, creating significant procedural and logistical challenges that limit its wider adoption.^7^ Additionally, in clinics that use BRT, it is important to recognize that this procedure depends on the operator and requires specialized expertise. Improper placement or displacement of applicators can decrease local control and disease‐free survival rates.^8^ Furthermore, BRT may cause side effects such as uterine perforation, vaginal laceration, and anesthesia‐related risks.^9^ Lastly, some patients may refuse applicator insertion due to anxiety or discomfort.^10^

The simultaneous integrated boost (SIB) plan is one of the EBRT methods that can be utilized as an alternative to BRT in patients with cervical cancer.^6^ The SIB plan allows for the irradiation of all target volumes throughout the treatment period, delivering different dose levels to various target volumes simultaneously.^11^ Additionally, when using this method, it is recommended that the dose evaluation be interpreted from a radiobiological perspective.^12^ Due to recent advancements in technology, SIB treatment plans can now be effectively implemented using external volumetric modulated arc therapy (VMAT) and helical tomotherapy (HT) techniques. Moreover, the integration of image‐guided radiation therapy (IGRT) ensures the precision and reproducibility of these procedures, thereby enhancing treatment accuracy.

This retrospective exploratory study evaluated the dosimetric feasibility of SIB in patients with locally advanced cervical cancer who were ineligible for BRT through a comparative analysis of VMAT‐ and HT‐based SIB plans.

## 2 MATERIALS AND METHODS

### 2.1 Patient selection

Ethical approval for this study was obtained from the institutional Clinical Research Ethics Committee, and all procedures were conducted in accordance with the Declaration of Helsinki. We performed a retrospective exploratory analysis of 10 patients with locally advanced cervical cancer treated with concurrent chemoradiotherapy followed by BRT between 2019 and 2021. The study complied with relevant Equator guidelines, ensuring methodological transparency and reporting standards.^13^ All patient data were anonymized to protect confidentiality. Patients were staged according to the FIGO staging system based on clinical evaluations and imaging studies.^14^

### 2.2 Patient preparation and computed tomography (CT) simulation scanning

Computed tomography (CT) images were obtained using a GE Discovery RT simulation device (GE Healthcare, USA). According to our clinical protocol, patients were asked to empty their rectum and fill their bladder before imaging. To keep patients stable during the procedure, a T‐board and knee and foot positioners were used. After these preparations, a CT scan was performed with a slice thickness of 2.5 mm, with patients positioned supine and head‐first.

### 2.3 Contouring

The target volume and OAR were contoured following consensus guidelines.^15^ The primary tumor was outlined as the gross tumor volume (GTVp), and the metastatic lymph node was outlined as the GTVn. The clinical target volume (CTV) included the GTVp, GTVn, cervix, uterus, and proximal vagina, based on physical examination and radiological imaging, which considered tumor invasion extent, along with the parametrial tissue and elective lymphatic regions. The elective lymphatic regions covered the pelvic (external iliac, internal iliac, obturator, presacral, and common iliac), para‐aortic, and inguinal lymphatic drainage areas, tailored to the patient's specific condition. For the elective 45 Gy volume, the corresponding planning target volume (PTV) was generated by isotropically expanding the CTV by 5 mm in all directions to compensate for setup errors and organ motion. PTV77.5 was defined as GTVp with no additional margin (0 mm), emphasizing conformity and OAR sparing, assuming daily soft‐tissue IGRT with cone‐beam computed tomography (CBCT). The main OARs delineated include the bladder, rectum, sigmoid, bowel, and femoral heads.

### 2.4 Radiotherapy dose and organs at risk

A study by Guerrero et al. assessed the use of SIB as an alternative to BRT. In this research, the target volumes received 45 and 77.5 Gy in 25 fractions using the SIB plan. This method was determined to be radiobiologically equivalent to the traditional approach of 45 Gy EBRT in 25 fractions plus 30 Gy in 5 fractions of High‐Dose‐Rate (HDR) BRT.^12^

When evaluating doses to OARs, criteria established for standard fractionation were used.^17^ To improve accuracy, OAR doses were converted to equivalent dose in 3.1 Gy fractions (EQD3.1) using a fraction size of 3.1 Gy and an α/β of 3, as shown in Equation (1); the converted values are listed in Table 1. *D* = total dose, *d* = dose per fraction, *α* = linear (first‐order dose‐dependent) component of cell killing, β = quadratic (second‐order dose‐dependent) component of cell killing, *α*/*β* ratio = the dose at which both components are equal^16^

(1) $$\mathrm{EQD}\;3.1_{\alpha /\beta }=\;D\;\left[\frac{d+\alpha /\beta }{3.1+\alpha /\beta }\right]$$

**TABLE 1:** Dose constraints for organs at risk, recalculated using EQD2 and EQD3.1 models (*α*/*β* = 3 Gy).

OAR	DVH Metric		Volume
	EQD2	EQD3.1	
Bladder	V80 Gy	V65.57 Gy	< 15%
	V75 Gy	V61.48 Gy	< 25%
	V70 Gy	V57.38 Gy	< 35%
	V65 Gy	V53.28 Gy	< 50%
Rectum	V75 Gy	V61.48 Gy	< 15%
	V65 Gy	V53.28 Gy	< 25%
	V60 Gy	V49.18 Gy	< 35%
	V50 Gy	V40.98 Gy	< 50%
Femur Head	V50 Gy	V40.98 Gy	< 5%
Bowel	V45 Gy	V36.89 Gy	< 195 cc

Abbreviations: DVH, dose–volume histogram; EQD, equivalent dose; OAR, organ‐at‐risk.

(**EQD3.1 dose conversion**).

### 2.5 Treatment planning

#### 2.5.1 VMAT planning

VMAT technique‐based SIB plans were created using the Monaco version 5.51 treatment planning system (TPS) (Elekta, Stockholm, Sweden). The SIB plans prescribed a total dose of 77.5 Gy in 25 fractions (3.1 Gy per fraction) to the PTV77.5, while the PTV45 received 45 Gy in 25 fractions (1.8 Gy per fraction). A 6 MV flattening filter‐free (FFF) beam was used. Two arcs were planned with gantry angles rotating clockwise from 180° to 180°. The gantry increment was set to 15°. Calculation parameters included a voxel spacing of 0.3 cm, a medium for dose deposition, and the Monte Carlo algorithm for dose calculation. The statistical uncertainty was set at 1%, and the minimum segment width was chosen as 0.5 cm. The goal was for at least 95% of the target volumes to receive 95% of the prescribed dose. Dose constraints specified in Table 1 for critical organs, as well as the D2cc maximum dose limits outlined in Table 2, were aimed to be respected.^17^, ^18^ Additionally, it was ensured that the maximum dose, defined as 107% of the prescribed dose, did not exceed 2 cc.

**TABLE 2:** D_2cc_ values for organs at risk based on EQD2 and EQD3.1 (*α*/*β* = 3 Gy).

OAR	EQD2		EQD3.1	
	Optimal (Gy)	Mandatory (Gy)	Optimal (Gy)	Mandatory (Gy)
Bladder	< 80	< 90	< 65.57	< 73.77
Rectum	< 65	< 75	< 53.28	< 61.48
Sigmoid Colon	< 70	< 75	< 57.38	< 61.48
Bowel Bag	< 70	< 75	< 57.38	< 61.48

Abbreviations: EQD, equivalent dose; OAR, organ‐at‐risk.

#### 2.5.2 Tomotherapy planning

HT technique‐based treatment plans were generated using the Precision version 3.3.1 planning system (Accuray, Madison, Wisconsin, USA) with 6 MV flattening filter‐free (FFF) photon beams delivered by the TomoTherapy HDA unit. In the planning system, the jaw mode was set to Dynamic, with a field width of 2.5 cm, a pitch factor of 0.287, and a modulation factor of 2–4. The optimization resolution was set to medium, while the final dose calculation resolution was set to high. The prescribed doses in HT‐based SIB plans were consistent with those in VMAT‐based SIB plans. Target volume coverage and OAR dose constraints were aimed to be comparable between the two techniques.

### 2.6 Statistical analysis

Statistical analyses were performed using IBM SPSS Statistics version 23.0 (IBM Corp., Armonk, NY, 2016). Descriptive statistics summarized baseline characteristics. The normality of data distribution was assessed with the Kolmogorov–Smirnov and Shapiro–Wilk tests. For variables that were not normally distributed, the Wilcoxon matched‐pairs signed‐rank test was used, while the paired sample t‐test was applied to normally distributed data. P values less than 0.05 were considered statistically significant.

## 3 RESULTS

The study included a total of 10 patients with locally advanced cervical cancer. The median age was 57 years (range: 36–69). Histologically, seven patients were diagnosed with squamous cell carcinoma, and three had adenocarcinoma. Eight patients were HPV‐positive, while two were HPV‐negative. At diagnosis, four patients were staged as IIIC1, three as IIB, two as IVA, and one as IIA. The median PTV77.5 and PTV45 volumes were 83.4 cc (range: 14.9–218.2 cc) and 1188.4 cc (range: 1027.8–1898.5 cc), respectively. Baseline patient and treatment characteristics are detailed in Table 3.

**TABLE 3:** Patient and treatment characteristics.

Patient	Age	Histopathology	HPV	Stage	PTV77.5	PTV45
1	59	Adenocarcinoma	Positive	IIB	93.3 cc	1199.9 cc
2	47	Squamous cell carcinoma	Positive	IIIC1	14.9 cc	1177.0 cc
3	61	Squamous cell carcinoma	Positive	IVA	24.5 cc	1131.2 cc
4	38	Squamous cell carcinoma	Positive	IVA	206.8 cc	1660.6 cc
5	59	Squamous cell carcinoma	Positive	IIB	60.0 cc	1063.5 cc
6	62	Squamous cell carcinoma	Negative	IIIC1	73.6 cc	1027.8 cc
7	47	Adenocarcinoma	Positive	IIA	130.0 cc	1045.3 cc
8	69	Squamous cell carcinoma	Negative	IIB	59.9 cc	1898.5 cc
9	36	Adenocarcinoma	Positive	IIIC1	113.6 cc	1271.4 cc
10	55	Squamous cell carcinoma	Positive	IIIC1	218.2 cc	1355.4 cc

Abbreviations: HPV, human papilloma virus; PTV, planning target volume.

The comparison of dose metrics, conformity index (CI), and homogeneity index (HI) values for PTV45 and PTV77.5, derived from VMAT and HT technique‐based SIB plans, is shown in Table 4. For PTV45, the HT technique‐based SIB plans demonstrated D95% and D98% values that were significantly closer to the prescribed dose compared to the VMAT technique‐based SIB plans (*p* < 0.001 and *p* = 0.004, respectively). However, Dmean and CI for PTV45 did not show significant differences between the plans. For PTV77.5, Dmean and conformity index (CI) of the HT technique‐based SIB plans were significantly better than those of the VMAT technique‐based SIB plans, with *p*‐values of 0.001 and < 0.001, respectively. Nonetheless, the D95% and D98% values for PTV77.5 did not differ significantly between the two plans.

**TABLE 4:** Comparison of dose, conformity index (CI), and homogeneity index (HI) values of PTV45 and PTV77.5 obtained from VMAT and HT treatment plans.

Mean ± SD	VMAT	HT	*p*
PTV45			
V_95%_ (%)	96.49 ± 1.45	98.66 ± 1.15	0.004
D_95%_ (Gy)	43.20 ± 0.49	44.65 ± 0.81	<0.001
D_98%_ (Gy)	41.95 ± 0.91	43.51 ± 1.11	0.004
D_2%_ (Gy)	77.82 ± 1.34	79.81 ± 2.72	0.017
D_max_ (Gy)	82.27 ± 0.81	82.70 ± 1.20	0.394
D_mean_ (Gy)	50.22 ± 4.97	50.66 ± 2.06	0.720
D_min_ (Gy)	30.72 ± 3.84	30.63 ± 7.01	0.970
CI	0.77 ± 0.04	0.75 ± 0.06	0.130
HI	0.77 ± 0.03	0.77 ± 0.05	0.953
PTV77.5			
V_95%_ (%)	98.37 ± 1.59	98.08 ± 2.01	0.575
D_95%_ (Gy)	75.28 ± 1.06	76.15 ± 2.11	0.139
D_98%_ (Gy)	74.19 ± 1.55	74.74 ± 2.64	0.386
D_2%_ (Gy)	79.71 ± 0.74	81.75 ± 1.20	0.001
D_max_ (Gy)	82.18 ± 0.83	82.70 ± 1.20	0.324
D_mean_ (Gy)	77.54 ± 0.62	79.52 ± 1.43	0.001
D_min_ (Gy)	65.57 ± 5.09	67.80 ± 4.77	0.787
CI	0.71 ± 0.10	0.81 ± 0.10	<0.001
HI	0.07 ± 0.02	0.08 ± 0.03	0.07

Abbreviations: CI, conformity index; D2%, represent the doses received by 2% volumes of the PTV; D95%, represent the doses received by 95% volumes of the PTV; D98%, represent the doses received by 98% volumes of the PTV; Dmax, represent the maximum dose received by the PTV; Dmean, represent the mean dose received by the PTV; Dmin, represent the minimum dose received by the PTV; HI, homogeneity index; HT, helical tomotherapy; PTV, planning target volume; SD, standard deviation; V95%, represent volumes receiving 95% of the PTV doses; VMAT, volumetric modulated arc therapy.

**FIGURE 1:**
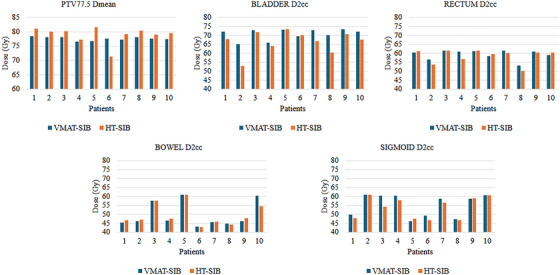
Patients PTV77.5 D_mean_ and OAR D_2cc_ doses. D_2cc_, the dose received by 2 cc of the organ; D_mean_, represents the mean dose received by the PTV; OAR, organ‐at‐risk; PTV, planning target volume.

The dosimetric comparison of OARs between VMAT and HT treatment plans is presented in Table 5. Both methods met the specified dose constraints for all OARs. HT showed superior OAR dose sparing compared to VMAT. Specifically, for the bladder, the V53.28 Gy and D2cc in HT planning were significantly lower than in the VMAT plan (*p* = 0.006 and *p* = 0.013, respectively). However, no statistically significant difference was observed in rectal doses between the two plans. Regarding the dose to the bowel, the V36.89 Gy in the HT plan was significantly lower than in the VMAT plan (*p* = 0.038). Still, there was no significant difference between the plans for D2cc in both the bowel and sigmoid. Additionally, the mean beam‐on times for VMAT and HT were 3.37  ±  0.38 min and 10.98  ±  2.37 min, respectively (*p* = 0.005). Figure 1 shows a graphical representation of the Dmean for PTV77.5 and the D2cc doses for the OARs.

**TABLE 5:** Comparison of EQD3.1‐equivalent OAR doses between VMAT and HT plans (*α*/*β* = 3).

Mean ± SD	VMAT	HT	*p*
Bladder			
V53.28 Gy (%)	10.31 ± 4.25	5.66 ± 3.26	0.006
V57.38 Gy (%)	7.07 ± 3.29	3.67 ± 2.24	0.005
V61.48 Gy (%)	4.64 ± 2.23	2.46 ± 1.46	0.004
V65.57 Gy (%)	2.75 ± 1.70	1.20 ± 0.91	0.003
D_2cc_ (Gy)	70.60 ± 3.11	66.47 ± 6.20	0.013
Rectum			
V40.98 Gy (%)	26.30 ± 9.19	28.00 ± 9.80	0.435
V49.18 Gy (%)	12.25 ± 4.07	11.68 ± 5.28	0.630
V53.28 Gy (%)	8.07 ± 3.35	7.83 ± 3.98	0.738
V61.48 Gy (%)	2.96 ± 1.64	2.54 ± 1.53	0.235
D_2cc_ (Gy)	59.26 ± 2.74	58.41 ± 3.82	0.333
Bowel			
V36.89 Gy (cc)	115.13 ± 38.93	94.03 ± 31.51	0.038
D_2cc_ (Gy)	49.57 ± 7.01	49.49 ± 6.03	0.445
Sigmoid			
D_2cc_ (Gy)	55.16 ± 6.20	53.70 ± 5.98	0.093
Right femoral head			
V40.98 Gy (%)	0.26 ± 0.44	0.24 ± 0.52	0.575
Left femoral head			
V40.98 Gy (%)	0.38 ± 0.57	0.28 ± 0.59	0.499

Abbreviations: D2cc, the dose received by 2 cc of the organ; EQD, Equivalent Dose; HT, helical tomotherapy; SD, standard deviation; VMAT, volumetric modulated arc therapy; Vx, the percentage of organ receiving more or equal to x Gy.

**FIGURE 2:**
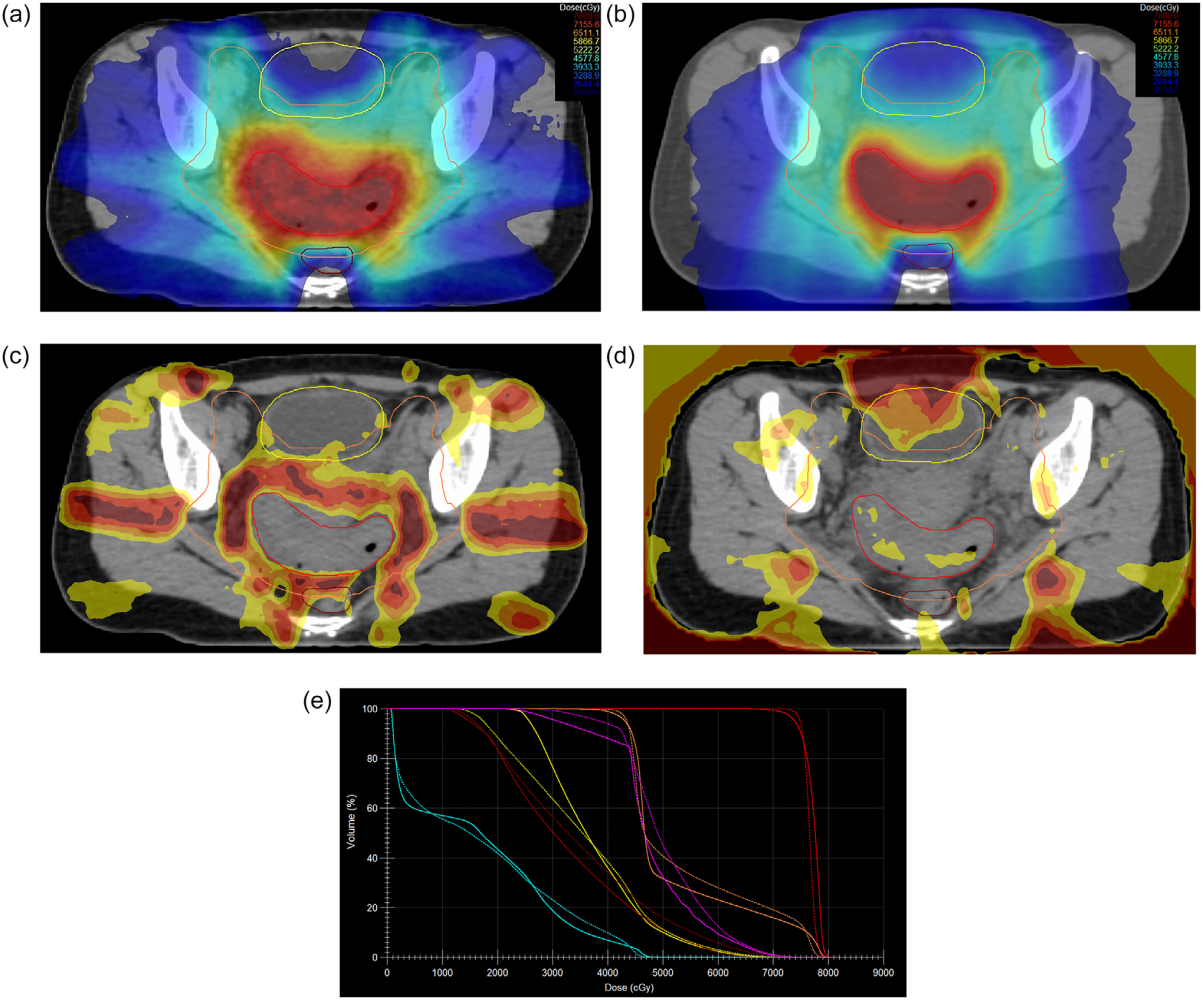
36‐year‐old female with HPV‐associated cervical squamous cell carcinoma (T4N1M0, stage IVA) treated with 45 Gy/25 fractions EBRT followed by 28 Gy/4 fractions brachytherapy. SIB plans and DVHs generated with VMAT and HT techniques. (a) VMAT‐based SIB plan dose distribution. (b) HT‐based SIB plan dose distribution. (c) Dose distribution of the VMAT minus HT SIB plan. Dose color wash: brown = 10 Gy; red = 7.5 Gy; orange = 5 Gy; yellow = 2.5 Gy. The following color codes indicate target volumes and OARs: orange = PTV45; red = PTV77.5; yellow = bladder; brown = rectum. (d) Dose distribution of the HT minus VMAT SIB plan. Dose color wash: brown = 10 Gy; red = 7.5 Gy; orange = 5 Gy; yellow = 2.5 Gy. The following color codes indicate target volumes and OARs: orange = PTV45; red = PTV77.5; yellow = bladder; brown = rectum. (e) DVHs of SIB plans generated with VMAT and HT techniques. Solid lines represent VMAT‐SIB and dashed lines represent HT‐SIB. The following color codes indicate target volumes and OARs: orange: PTV45; red: PTV77.5; yellow: bladder; brown: rectum; light blue: bowel; pink: sigmoid. DVH, dose–volume histogram; EBRT, external beam radiotherapy; HPV, human papillomavirus; HT, helical tomotherapy; OAR, organ‐at‐risk; PTV, planning target volume; SIB, simultaneous integrated boost; VMAT, volumetric modulated arc therapy.

As shown in Figure 2, a 36‐year‐old woman evaluated for abnormal vaginal bleeding was diagnosed with HPV‐associated cervical squamous cell carcinoma invading the bladder and bilateral parametrium, with a metastatic lymph node in the left internal iliac area. She received concurrent cisplatin‐based chemoradiotherapy with EBRT (45 Gy in 25 fractions) followed by BRT (28 Gy in 4 fractions). The PTV77.5 volume was 206 cc, making it the second largest in the cohort. The volume receiving 95% of the prescribed dose was 99.08% with VMAT and 96.8% with HT; no volume received 107%. Dmin, Dmean, and Dmax for PTV77.5 were 68.7, 76.54, and 81.47 Gy with VMAT, and 63.89, 77.28, and 80.6 Gy with HT. D2cc doses to the bladder, rectum, bowel, and sigmoid were 65.77, 60.85, 46.50, and 60.25 Gy for VMAT, and 63.91, 56.58, 47.6, and 57.69 Gy for HT.

## 4 DISCUSSION

In this study, SIB plans were re‐generated using the VMAT and HT techniques in patients with locally advanced cervical cancer who had undergone BRT following EBRT. The study compared the two techniques and assessed the dosimetric feasibility of SIB in BRT‐ineligible patients. Overall, compared to VMAT technique‐based SIB plans, HT technique‐based SIB plans demonstrated superior target dose coverage while concurrently achieving improved dose sparing for OARs such as the bladder and bowel. The only advantage of the VMAT technique over HT was its shorter treatment delivery time. Both VMAT and HT technique‐based SIB plans have demonstrated potential as alternatives for patients with locally advanced cervical cancer in whom BRT cannot be applied.

BRT plays a crucial role in treating cervical cancer. Numerous studies have shown that BRT following EBRT improves overall survival and local control. As a result, current treatment guidelines strongly recommend using BRT in non‐surgical cervical cancer cases. However, challenges such as training, cost, and the need for sedation remain. A 2023 study by Williamson and Mayadev highlighted access issues and the underuse of BRT in the United States.^19^ Consequently, alternative treatments to BRT have been explored. A comprehensive review examined the role of Stereotactic Body Radiotherapy (SBRT) boost after EBRT in patients who cannot receive BRT, reporting promising local control rates.^20^ Additionally, a study compared BRT plans with those generated using CyberKnife, an SBRT technique, in cervical cancer patients. The comparison of the CTVs showed that CyberKnife‐based plans offered better target coverage and OAR sparing.^21^

There is a substantial body of literature on the use of the SIB technique in cervical cancer. In one study involving radiologically node‐positive cervical cancer patients, it was shown that delivering 60 Gy in 25 fractions to the lymph node did not result in a significant increase in toxicity.^22^ In another study, the 5‐year outcomes of SIB to positive lymph nodes in node‐positive cervical cancer patients demonstrated promising treatment responses and low toxicity rates.^23^ When studies focusing on SIB applied to the primary tumor are examined, one study reported that in cervical cancer patients treated with neoadjuvant VMAT delivering 50.6 Gy to the primary tumor in 25 fractions, the pathological complete response rate was 46% and the 3‐year overall survival (OS) rate was 89.0%.^24^ In another study, Intensity‐Modulated Radiation Therapy with SIB (IMRT‐SIB) and Three‐Dimensional Conformal Radiation Therapy with Sequential Boost (3DCRT‐SB) plans were compared in cervical cancer patients, and IMRT‐SIB was found to achieve lower OAR doses.^25^

A review of the literature shows that studies exploring the use of the SIB plan as an alternative to BRT are limited. In a study involving cervical cancer patients who did not undergo BRT, a Biologically Effective Dose (BED) of 83.3 Gy (*α*/*β* = 10) was delivered to the primary tumor using the SIB plan in patients with tumors smaller than 5 cm. Patients with tumors larger than 5 cm and bilateral parametrial invasion received a BED of 98.21 Gy to the primary tumor. These radiotherapy plans were created using the RapidArc technique. In our study, the BED planned for the primary tumor was 101.53 Gy (*α*/*β* = 10). They reported that all 14 patients were alive at the time of analysis, with disease progression observed in only one patient. Unlike our study, their research was conducted in vivo and included an assessment of treatment‐related toxicity, which is an important strength. However, despite delivering a higher BED to the primary tumor, we achieved lower doses to critical organs. The evaluation of both HT and VMAT techniques provides valuable insights into the literature.^26^

In another study, Sukhikh et al. compared VMAT‐SIB and VMAT‐SB techniques in cervical cancer patients who could not receive BRT and were treated with VMAT‐SB. In that study, an equivalent dose in 2 Gy fractions (EQD2) of approximately 88 Gy was planned for the primary tumor, whereas in our study, the targeted EQD2 is 84.61 Gy (*α*/*β* = 10). Sukhikh et al. demonstrated that VMAT‐SIB could be a good alternative to VMAT‐SB because it has the potential to shorten treatment time and better spare critical organs. Compared with this study, our results show that both VMAT‐SIB and HT‐SIB plans provided better protection of the bladder and rectum.^27^ In a study conducted on geriatric cervical cancer patients who could not receive BRT and were treated with VMAT‐SIB for the primary tumor, a radiotherapy dose equivalent to 80.52 Gy BED (*α*/*β* = 10) was delivered to the primary lesion. The study highlighted that VMAT‐SIB could be a feasible alternative for curative treatment in geriatric cervical cancer patients because of its acceptable treatment outcomes and toxicity profiles.^28^ Nonetheless, SIB‐based external‐beam boosts should mainly be considered an option for patients who are ineligible for BRT rather than as a routine replacement. EBRT followed by BRT remains the standard of care for the definitive management of locally advanced cervical cancer.

The EMBRACE II study, published in 2018, established treatment volumes and dose constraints for critical organs in cervical cancer through the development of Image‐Guided Adaptive Brachytherapy (IGAB). When comparing the total dose targeted to the primary tumor in terms of EQD2, EMBRACE aimed for 85–90 Gy, while in our study, the planned total dose to the primary tumor was an EQD2 of 83.3 Gy (*α*/*β* = 10). In setting dose constraints for critical organs, the limits defined by the EMBRACE II study were considered, with recalculations performed based on both EQD2 and EQD3.1. In all patients, these dose constraints were adhered to in both the VMAT‐SIB and HT‐SIB plans. Achieving target doses for the primary tumor and maintaining acceptable critical organ doses at this level with a non‐invasive technique, as an alternative to invasive procedures like BRT, is significant.^18^

BRT differs from EBRT by delivering high doses to small volumes through its steep dose fall‐off. The radiobiology of BRT remains complex. The effects of BRT can vary depending on the *α*/*β* ratio of the target tissue, the BRT technique used, and the radiation source applied. The Linear Quadratic (LQ) model is also used in BRT; however, the short treatment duration and high dose per fraction inherent to BRT introduce uncertainties.^29^ In SBRT, the use of high doses in a single fraction does not align with fundamental radiobiological principles such as reoxygenation and repopulation. Therefore, the LQ model may not accurately reflect treatment effectiveness at high fraction doses. As a result, the LQ model can generally be applied more reliably in SIB than in SBRT or BRT.^30^, ^31^

This study has several limitations. First, the small sample size and single‐center design limit the generalizability of the results. Second, as an in vitro dosimetric study, it does not provide data on clinical outcomes such as treatment response, survival, or toxicity. Third, given the study's retrospective, planning‐only design, serial anatomical changes, adaptive target reduction (shrinking), and multiple replanning steps were not simulated. Fourth, as IGRT has become more widely used, the literature has progressively reduced recommended CTV‐to‐PTV margins, which can increase target conformity and, in turn, reduce OAR doses. In this planning study, we therefore adopted a 0 mm boost margin (GTVp = PTV77.5) based on daily soft tissue IGRT and the option of adaptive replanning if target changes were observed. Nonetheless, compared with BRT, a zero margin in EBRT may still underestimate the risk of geometric miss. We also acknowledge that in low‐ and middle‐income countries, where access to daily IGRT or advanced imaging may be limited, the likelihood of such geometric miss is higher. Finally, BRT for this cohort was delivered at an outside institution, and patient‐level dosimetric data were not available. As a result, we could not perform a direct, parameter‐matched comparison with our EBRT‐SIB plans. To address this gap, we recommend conducting multicenter studies that collect comprehensive BRT dosimetric metrics and enable robust head‐to‐head evaluation.

## 5 CONCLUSIONS

BRT following EBRT is essential in the treatment of cervical cancer. When BRT cannot be administered due to patient‐related or other factors, SIB therapy offers a practical alternative. Of the SIB plans, HT provides greater advantages over VMAT because of better OAR sparing and enhanced target coverage. However, for SIB to be integrated into treatment guidelines, multicenter prospective studies are necessary.

## AUTHOR CONTRIBUTIONS

Muhammed İsmet Sekmen contributed to manuscript writing, data collection, and statistical analysis. Timur Koca was responsible for study design and overall supervision. Durmuş Ali Çetmi, Rahmi Atil Aksoy, and Aylin Fidan Korcum contributed to manuscript writing and literature review. Yılmaz Bilek, İsmail Hakkı Sarpün, and Nina Tunçel contributed to study design adjustment and compilation of statistical data. All authors reviewed and approved the final version of the manuscript and agree to be accountable for all aspects of the work.

## CONFLICT OF INTEREST STATEMENT

The authors declare that they have no conflict of interest.

## ETHICAL APPROVAL

This study was approved by the Akdeniz University Faculty of Medicine Clinical Research Ethics Committee on 29.02.2024 (Decision number: 158).

## Data Availability

Research data are stored in an institutional repository and will be shared upon request to the corresponding author.
